# How Phantom Networks, Provider Qualities, and Poverty Sway Medicaid Dental Care Access: A Geospatial Analysis of Manhattan

**DOI:** 10.3390/ijerph182312383

**Published:** 2021-11-25

**Authors:** Destiny Kelley, Shipeng Sun

**Affiliations:** 1 Department of Geography and Environmental Science, Hunter College, CUNY, 695 Park Avenue, New York, NY 10065, USA; destiny.kelley78@myhunter.cuny.edu; 2 Earth and Environmental Sciences Program, Graduate Center, CUNY, 365 Fifth Avenue, New York, NY 10016, USA

**Keywords:** phantom networks, hidden networks, Medicaid, managed care organization, general dentistry, two-step floating catchment area method

## Abstract

Access to general dental care is essential for preventing and treating oral diseases. To ensure adequate spatial accessibility for the most vulnerable populations, New York State mandates a ratio of one general dentist to 2000 Medicaid recipients within 30 min of public transportation. This study employed geospatial methods to determine whether the requirement is met in Manhattan by verifying the online directories of ten New York managed care organizations (MCOs), which collectively presented 868 available dentists from 259 facilities. Our survey of 118 dental facilities representing 509 dentists revealed that significantly fewer dentists are available to treat Medicaid recipients compared to MCO directories. The average dentist-to-patient ratio derived from the MCO listings by the Two-Step Floating Catchment Area (2SFCA) method was 1:315, while the average verified ratio was only 1:1927. “Phantom networks”, or inaccurate provider listings, substantially overstated Medicaid dental accessibility. Surprisingly, our study also discovered additional Medicaid providers unlisted in any MCO directory, which we coined “hidden networks”. However, their inclusion was inconsequential to the overall dental supply. We further scrutinized dental care access by uniquely applying six “patient-centered characteristics”, and these criteria vastly reduced accessibility to an average ratio of merely 1:4587. Our novel evaluation of the spatial association between poverty, dental care access, and phantom networks suggests that Medicaid dental providers wish to be located in wealthier census tracts that are in proximity to impoverished areas for maximum profitability. Additionally, we discovered that poverty and phantom networks were positively correlated, and phantom providers masked a lack of dental care access for Medicaid recipients.

## 1. Introduction

Access to dental care is critical to maintain both oral and overall health. Research links poor oral health to a multitude of diseases, such as diabetes [1], arthritis [2], cancer [3], and cardiovascular disorders [4,5,6]. The occurrence of periodontitis and gum inflammation, for example, is among the most frequent causes of tooth loss [7], and possessing fewer teeth is correlated to a shorter life expectancy [8]. Remarkably, the incidence of dental caries is virtually preventable with proper dental care [9], and long-term clinical studies have shown that periodontitis can also be inhibited utilizing dentistry interventions [10,11,12].

In developed countries, dental disease more often affects socioeconomically disadvantaged populations [13]. In the U.S., inequalities in untreated cavities are historically higher among Hispanic and Black adults, with rates of poverty playing a key role in access to oral healthcare [14]. Medicaid recipients rely on state-contracted managed care organization (MCO) provider directories as a starting point in obtaining access to dental care. While MCO listings provide a useful source concerning the supply side of dental care, the lists may be flawed, outdated, and contain inaccurate data. Even when the listed providers are available, their services might be limited due to office hours, service caps, and other factors.

Utilizing the multifaceted “Access framework” for general healthcare as reconceptualized from Lipson et al. [15], this paper examines Medicaid recipients’ dental care access issues, with a focus on the dimensions of availability, accessibility, and accommodation among MCO listed providers. In terms of availability, healthcare providers inaccurately listed as participating in an insurance plan constitute “phantom networks” [16,17]. For over two decades, the phenomena of phantom networks and inaccurate insurance provider directories were found across numerous geospatial locations and multiple medical disciplines, dispersed among both private and public health organizations [16,17,18,19,20,21,22,23,24]. However, there is a lack of research about dental phantom networks, and this study will explore and expand the topic to better understand this multidimensional phenomenon.

Past research has predominantly focused on calculating the Medicaid dental supply utilizing provider enrollment as the primary determinant for evaluating access to publicly funded dental care, without inspecting other utilization metrics [25,26]. Specifically, we define “patient-centered” dental facilities as those that provide extended office hours, where more than half of the dentists working at the facility accept new patients, offer a full-range of services, place no caps on the number of Medicaid patients seen during any time frame, work full-time, and serve all age groups. Most of the patient-centered criteria used in this study are derived from the conceptualized framework of Lipson et al. [15].

However, the criterion for caps on the number of Medicaid patients treated stems from various literature [27,28,29,30], while the range of dental services is a novel topic that this study incorporated to ascertain the overall care available to Medicaid patients. Some dental facilities may limit the range of services available to Medicaid patients, even if those services are covered by Medicaid [31]. This practice is permissible in New York State since there is no restriction under dental licensure requirements [32] nor in the state’s dental policy and procedure manual [33].

Several prior studies have examined a few aspects of structural supply elements under the dimension of availability, including the acceptance of new patients [34,35,36,37], dental provider working status [37,38,39,40,41,42], and the age range of groups treated [34,35,36,37,43]. Nevertheless, to the best of our knowledge, no research to date has addressed whether participating dentists limit the range of services for Medicaid patients, and how many providers place caps on the number of publicly insured patients that they are willing to treat during any timeframe.

Furthermore, under the dimension of accommodation, research has suggested that extended office hours may have the potential to increase dental care utilization [44] and deter costly non-traumatic emergency room visits [45,46]. Although one academic study [47] and two financial group whitepapers [48,49] cited a patient preference for expanded dental care working hours, there remains little mention of extended office hours within the literature. This study uniquely defines a full range of dental supply elements under the term “patient-centered characteristics”, and then applies spatial methods to discover their effect upon accessibility in a large metropolitan area.

Within the access framework, accessibility concerns the spatial location or proximity of providers to patients in terms of geographical travel time and distance. Numerous dental care accessibility studies have utilized area-based provider-to-population ratios [25,39,50,51,52,53,54] and distance-based measurements [55,56,57,58,59,60], which fail to account for “spatial accessibility”, a measure of both availability and accessibility. The two-step floating catchment area method (2SFCA) is a spatial analysis method designed to tackle this issue [61,62,63], and it has been widely applied in healthcare access studies along with various modifications during the last decade [64]. Conversely, the original 2SFCA has only recently been applied to dental care access research in the U.S. [40,42].

Although dental coverage is not implemented in the federal Medicaid program, nineteen states, including New York (NY), offer extensive dental benefits to their publicly insured adult recipients [65]. New York State (NYS) mandates that 2000 Medicaid recipients must have access to at least one general dentist (1:2000) within thirty minutes of public transportation [66,67]. The setting for this study is the metropolitan area of Manhattan, and it is one of the five boroughs that make up New York City (NYC). As of 2019, the population of Manhattan was estimated to be 1.6 million people, with a poverty rate of 15.6% [68], which is greater than the rate for both NYS at 13.6% [69] and the nation at 11.8% [70]. In NYC, Black and Latino New Yorkers are disproportionately concentrated in higher-poverty neighborhoods compared to white or Asian Americans [71].

Several studies have found that disparities regarding dental care access are more likely to occur in areas with higher poverty rates or lower incomes [51,57,58,72,73]. However, none of the reports focused solely on Medicaid dentists, nor did they examine the influence of location on the association between dental care access and poverty rates. This research is novel and will attempt to establish whether geographic areas with the greatest proportion of poverty suffer disparities from a lack of Medicaid dental providers. The objectives of this study were to (1) measure spatial access to general dental care for Medicaid beneficiaries in Manhattan by comparing the directory data of ten MCOs to verified Medicaid providers; (2) probe participation in patient-centered dental practice characteristics that may affect patient access to the dental supply; (3) quantify the spatial association between poverty rates and dental care access ratios; and (4) examine the relationship between neighborhood poverty rates and phantom networks to uncover disparities in access to dental care.

## 2. Materials and Methods

### 2.1. Data Collection and Processing

First, the contents and accuracy of Medicaid MCO online dental directories were assessed to determine Medicaid dental care access. There are ten mainstream Medicaid Managed Care Plans, and they were retrieved as of October 2019 from the Medicaid Data Warehouse, which provides insurance for Manhattan’s Medicaid recipients (Table 1). After examining the directories, the MetroPlus Medicaid Managed Care and MetroPlus Special Needs plans were grouped together since the dental providers were the same for both. While most of the respective MCO websites contained their own dental directories, a few redirected to third-party dental administrator organizations: AmidaCare to Healthplex, Empire Blue Cross Blue Shield to Liberty Dental, and Emblem Health to DentaQuest.

A series of survey questions were formulated for the listed Medicaid dental providers to test the accuracy of Medicaid MCO directories, and these questions are presented in the Supplementary Materials. The survey questions comprised the following: (1) the dental facility location and operating hours; (2) whether the facility provided general dentistry services; (3) whether any of the ten Medicaid MCO insurance plans were accepted; (4) new patient acceptance by insurance plan; (5) the age groups of patients served; (6) the range of services offered; (7) any caps on the number of patients seen; and (8) the working status of the verified dentists. The survey was conducted by the presenting author. Respondents who were allowed to answer the survey questions included dentists or any other authorized staff who possessed knowledge of facility services, such as managing appointments, scheduling, and/or billing.

Interaction with dental facilities was in the form of telephone calls, email communications, and an internet survey using Qualtrics survey software from November to December of 2019. Dental facilities without emails were contacted by phone and were also given the option to complete the survey online. Dental facilities with available email addresses were first contacted electronically and asked to complete the survey through Qualtrics, as well as given the option to complete the survey by telephone. If the emailed Qualtrics’ questionnaire remained completely unanswered after ten business days, then the facility was contacted by phone. Consent was obtained by all respondents.

Participating dental providers were classified into three categories: verified dentists, phantom providers, and eliminated dentists. By the criteria of this study, verified dentists serve either “adults and children” or “adults only”. Additionally, facilities serving only adults were still counted as general providers because access for this population is more limited, as Medicaid dentists may prefer to serve children [35,37,74]. Daw [16] originally defined phantom networks as a group of providers not participating in an insurance plan or no longer accepting new patients under the plan. We modify and expand this definition to include multiple errors in MCO directories of available providers that overstate access to care. We define the criteria for this category as mislabeled addresses, closed offices, unreachable facilities, a lack of general dentistry services, an absence of services provided by licensed dentists, refusal to accept Medicaid, and dental services provided to a limited population.

Dentists that provide onsite services at primary and secondary schools were designated as phantom providers given that they only serve the limited population of children who are enrolled at the specific school site. Dentists who only treat institutionalized nursing home patients were also assigned phantom status as they do not treat the general population of Medicaid recipients. However, we do not classify dentists no longer accepting new patients as phantom networks since this could unfairly reduce the supply of dentists who still treat current patients on behalf of the Medicaid community.

Eliminated dentists included providers in regular practice who serve only children. Per our study criteria, dentists working at a single facility less than 20 h per week were likewise classified as eliminated, since they failed to provide at least half-time oral healthcare based on a full-time equivalent (FTE) status of a 40-h work week, thus diminishing their impact on behalf of Medicaid recipients. Moreover, there is no uniform standard for dental work status in the literature; however, most studies, including those of the ADA, have defined full-time equivalent (FTE) status as 32 h or more per week [37,38,40,41,42,53,54,75], and some studies have excluded non-FTE dentists when calculating the supply of providers [40,42,53,54,75]. This study chose a modest half-time work status to allow for the greatest inclusion of available dental care access.

After initial data collection, we found that some dental facility names and dental providers were repeated more than once across all insurance lists, in part because many dentists accepted more than one Medicaid insurance. When duplicate facilities, defined by unique addresses, and repeating dentist names were removed, a total of 601 dentists and 259 dental facilities each accepted at least one mainstream Medicaid insurance plan based on the combined insurance lists. However, the 259 dental facilities collectively comprised 868 dentists, due to a significant number of providers listed as working at multiple locations, and this was the total number included in the geospatial analysis for MCO-listed dentists.

All provider numbers that are displayed in this study include duplicate dentists working at multiple facilities rather than the distinct number of dentists. This method reflects the idea behind the two-step floating catchment area method, in which duplicate dentists are counted as active providers at different facilities in different geographic locations for full representation. Further, eight facilities listed by Fidelis Care lacked dentist names in their directories, and these facilities did not occur in any other insurance list. Therefore, the number of Medicaid dentists working at these facilities was estimated using the average number of MCO-listed dentists working at surrounding facilities within a one-mile radius [76].

Our study chose to include non-respondents to avoid unfairly reducing the dental supply. Therefore, characteristics of non-respondent dental facilities were estimated from the known characteristics of respondent dental facilities across the entire borough for verified and patient-centered providers. Although this approach cannot accurately estimate the characteristics of these non-respondent facilities at the individual level, this method should statistically reflect their overall characteristics. Specifically, we used the random number weighted probability method to assign non-respondent characteristics [77]. Additionally, patient-centered dental facilities were extracted from verified dentists by filtering for facilities in which more than half of the dentists working at the facility accepted new patients, offered a full range of services, placed no caps on Medicaid patients seen, served all age groups, worked full-time, and provided extended hours. To be exact, these patient-centered dental facilities provide unconstrained services to Medicaid patients.

### 2.2. Statistical and Geospatial Analysis

To implement the analysis, we spatialized the addresses of the surveyed dental facilities, built a public transportation network, compiled poverty data from the census, and derived the Medicaid dental care access ratios. All 259 dental facility addresses were geocoded using ArcGIS Pro, which produced a 99% locale match. The remaining 1% contained a trivial difference in the building number, such as storefront instead of office number, which still corresponded to the correct location. Construction of the public transportation network was executed using LION street data and October 2019 General Transit Feed Specification (GTFS) files consisting of the MTA Metro-North railroad, bus routes, and subway lines [78,79,80].

For the poverty data, we chose the census tract as the spatial unit of analysis since it was the finest unit available for New York County. Census tracts with a total population of less than 1000 were excluded because they are primarily non-residential. Additionally, the entire residential population of Randall’s Island is institutionalized, and, as such, they are excluded from this study since this group does not represent the general non-institutionalized Medicaid recipient looking for dental care. For the remaining tracts, Total Population, Allocation of Medicaid/Means-Tested Public Coverage, and the Ratio of Income to Poverty Level in the past 12 months were acquired from the American Community Survey (ACS) 5-year estimates for years 2013 to 2017 [81]. As the number of Medicaid enrollees increased from approximately 205,000 in 2017 to 272,000 in September 2019 [82], we applied the same percentage of increase to each census tract, assuming even growth across Manhattan.

With these geospatial data, the 2SFCA was performed to calculate the Medicaid dentist-to-recipient ratios in census tracts [83]. These ratios, together with poverty data, were mapped, tested, and analyzed using non-spatial and spatial statistical methods. First, to determine whether these ratios met the state’s 1 to 2000 requirement with statistical significance, one-sample *t*-tests of the mean for these ratios were performed after they were found normally distributed [84,85,86]. To test the correlation between phantom networks and poverty within the same census tracts, non-parametric testing was necessary since the distribution of poverty was right-skewed. We therefore applied both Kendall’s tau and Spearman’s rho rank correlation tests for a more robust conclusion, although they typically yield similar results [87,88,89].

Lastly, we used various geospatial methods to examine the spatial association between poverty (denoted by the number of people with an income-to-poverty ratio under one in a census tract), dental access ratios, and phantom providers [90]. The spatial relationship between poverty and dental care access was measured in GeoDa [91] utilizing a multivariate Local Geary test with K-nearest neighboring census tracts, where we set K to six as it was roughly the average number (≈6.14) of contiguous neighbors for the tracts. Unlike the non-spatial global correlation tests, this spatial test considers the correlation of poverty and Medicaid dental access between census tracts and their neighbors instead of within the same tracts. Specifically, for this study, non-respondents were excluded to determine the greatest accuracy of actual phantom network clustering. Although some dental facilities were located in the excluded census tracts, all of them were within 700 feet of at least another tract boundary. Therefore, a spatial join was performed between the facilities containing phantom providers and the census tracts, encompassing all the facilities within 700 feet of each census tract to account for facilities within those excluded tracts. The optimized hot spot analysis was conducted using the poverty data and the number of phantom dentists and phantom facilities to discover any clustering or “hot spots” for these phenomena at a 90% confidence level or higher. We then identified locations where both poverty and phantom network hot spots occurred simultaneously. Global Moran’s I Spatial Autocorrelation reports were generated to discover the overall clustering or dispersion patterns of poverty numbers, count of phantom facilities, and sum of phantom dentists.

## 3. Results

### 3.1. The Verdict for MCO Directories

The MCO databases listed a total of 868 general dentists working at 259 facilities (Table 2). There were 118 facilities that responded to the survey, which represented 509 dentists as listed in MCO directories. The facility response rate was 45.6%, encompassing 58.6% of the listed providers. Quite often, survey phone calls were transferred to authorized staff having the best knowledge about the services provided. Each facility had its own organizational structure, and receptionists, assistants, or administrative personnel mainly responded to survey questions. At 37 facilities or approximately 30% of responding offices, dentists directly answered the questions, and this was primarily true for solo practices or those with less than three providers. Most individual dentists were designated as phantom providers, constituting 351 dentists or 69% of the providers, while 17.3% of dentists were eliminated, comprising 88 dentists. Merely 70 dentists were verified as Medicaid providers, which is 13.8% of the MCO-listed dentists.

The primary reason for elimination was due to dentists serving only children, comprising 49 providers (9.6%), while 39 dentists worked less than half-time (7.7%), as shown in Table 3. “Dentists do not work at listed facilities” was the primary reason that dental providers were deemed to be phantom networks, totaling 113 dentists (22.2%), as seen in Table 4. Dentists serving a limited population, consisting either of children enrolled at a specific school or nursing home residents, were the second-greatest cause of phantom status, comprising 92 dentists (18.1%). The third reason for phantom classification was that licensed dentists customarily did not treat any patients at the facility, consisting of 82 dentists (16.1%). Many of these providers merely supervised unlicensed dental school students treating patients, while a few dentists simply owned the practice, without providing patient care. As an aside, it was common for respondents to state that listed dentists had either left the practice or quit accepting Medicaid payments several years prior, while a small number of dentists never worked at the listed MCO facility, to the knowledge of office staff.

Interestingly, a fourth category of dental providers emerged over the course of this research. The phenomenon of a “hidden network” is a term coined by this study to describe the opposite of a “phantom network” (Table 2). It was found that six dentists did in fact accept MCO insurance plans, even though these data were unlisted in the plan directories. Notably, three directory dentists that refused the listed insurances, and were counted as phantom providers, did in fact accept at least one of the other nine Medicaid MCO plans, making the percent increase in verified dentists 12.9%. However, the addition of nine dentists as verified providers failed to significantly increase the Medicaid dental supply. Further information on breakdowns at the insurance plan level concerning dentist response rates and provider classification is included as Supplementary Materials online in Tables S1–S3.

**Figure 1:**
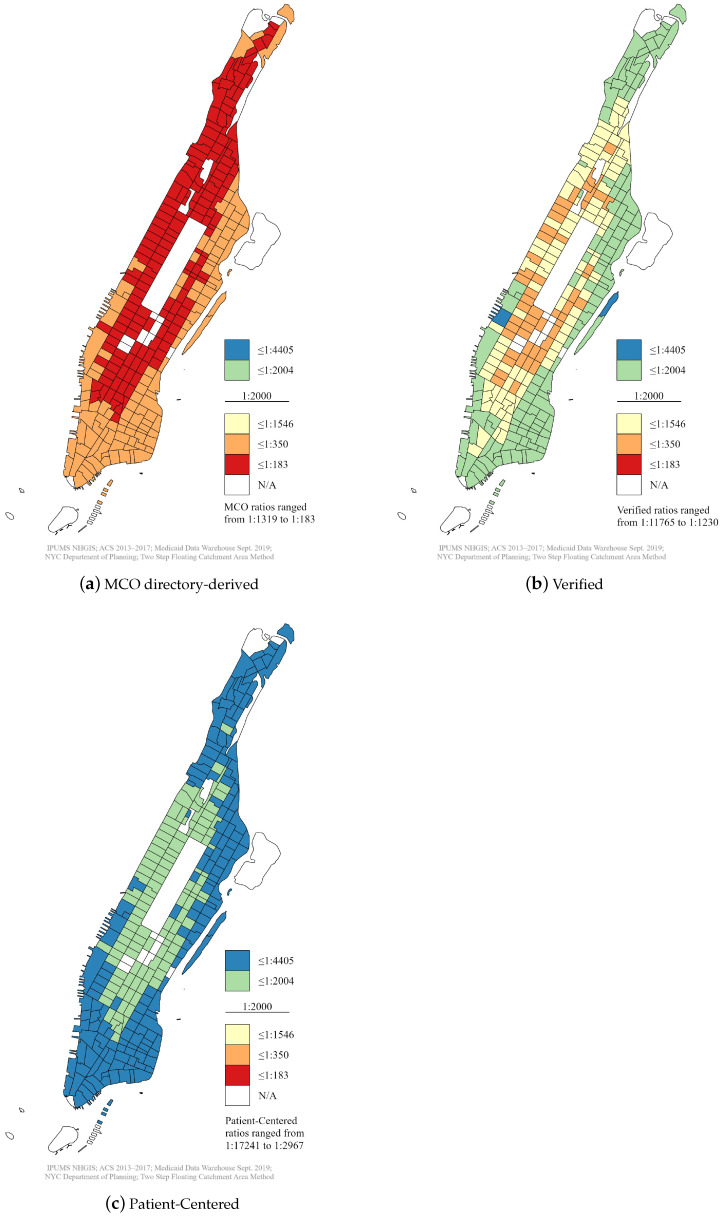
Medicaid dental supply in Manhattan census tracts, October 2019.

The supplied and unverified MCO directories provided a plethora of purported Medicaid dentists by the 2SFCA method (Figure 1). The state-mandated ratio of one dentist to 2000 Medicaid enrollees within 30 min of public transportation time was fulfilled well beyond the requirement in all cases. The one-sample *t*-test results additionally indicated that the state requirement was met (Table 5). However, the 2SFCA method confirmed that the supply of verified Medicaid dentists was significantly less than the MCO directories (Figure 1). After comparing the ratios spatially, 15–17% of the providers remained throughout the census tracts.

Nonetheless, the state requirement was still fulfilled in most census tracts, with the caveat that the average ratio for verified providers was much lower compared to dentists found in MCO directories. The one-sample *t*-test results confirmed that the state requirement was still fulfilled (Table 5). Notably, Figure 1 grouped all types of dental providers into the same range for better comparison between the maps; however, finer detail for the spatial distribution of each provider type was lost. Thus, Figure S1 is provided in the Supplementary Materials, where the individual details are preserved.

### 3.2. What Happened When Filtering for Patient-Centered Providers?

The supply of verified dentists was further reduced when filtering for patient-centered providers by the criteria of this study. The breakdown of these characteristics is computed in the aggregate using facility-level data for 50 offices (Table 6). Only one facility placed caps on the number of Medicaid patients treated (2%), and three facilities limited treatment to adults (6%). The great majority, or 46 facilities, accepted new patients and provided a full range of services, or 92% of the verified offices contacted. It should be noted that six facilities indicated that they periodically, for short intervals, stopped and then restarted accepting new patients. These facilities were still counted if they were accepting new patients when surveyed. Additionally, a significant portion of verified dentists worked 40 h or more per week at 35 facilities (70%), and 35 facilities offered extended office hours (70%). Of the facilities offering extended hours, 21 offices (60%) did not remain open more than one hour past 5pm on weekdays, while 20 offices (57%) were open at least one day during the weekend.

While a high percentage of dental facilities met at least one of the patient-centered characteristics, a mere 20 practices (40%) fully met all the applied conditions. The 2SFCA method established that the supply of patient-centered Medicaid dentists was substantially less than the verified dental supply, as confirmed by the one-sample *t*-test (Figure 1 and Table 5). As a result, the NYS-mandated ratio was no longer met. The resulting supply constituted a shortage of dentists in some census tracts according to the standard set by the U.S. Health Resources and Service Administration (HRSA), in which a ratio of one dentist to 5000 people is designated as a Health Professional Shortage Area (HPSA) [40,92].

### 3.3. Is Poverty Associated with the Medicaid Dental Supply?

**Figure 2:**
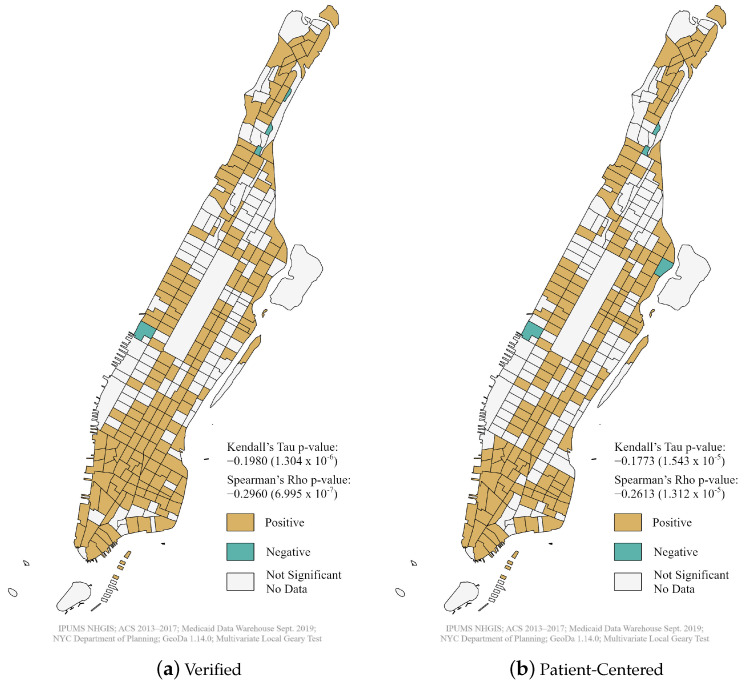
Multivariate Local Geary’s Test of dental supply and poverty in Manhattan census tracts, October 2019.

The Multivariate Local Geary test revealed a positive spatial association between dental care access for Medicaid recipients and poverty numbers in most Manhattan census tracts for verified and patient-centered dentists, especially in Southern Manhattan (Figure 2). A few census tracts displayed a negative relationship, and these were located either on the Upper West Side or in Upper Manhattan. However, the non-spatial Kendall’s and Spearman’s rank correlation tests provided different results from the spatial analysis.

Both tests found a weak negative relationship between dental care access and poverty numbers from verified and patient-centered ratios. However, the negative relationship derived from the Spearman’s rho values (−0.2960 and −0.2613) was stronger than the Kendall’s tau values (−0.1980 and −0.1773) for both verified and patient-centered dentists, respectively. The negative relationship was slightly less pronounced for patient-centered providers. As we will discuss later, these contrasting results have important implications for the locational decisions of Medicaid dental service providers.

### 3.4. Is Poverty Associated with Phantom Networks?

**Figure 3:**
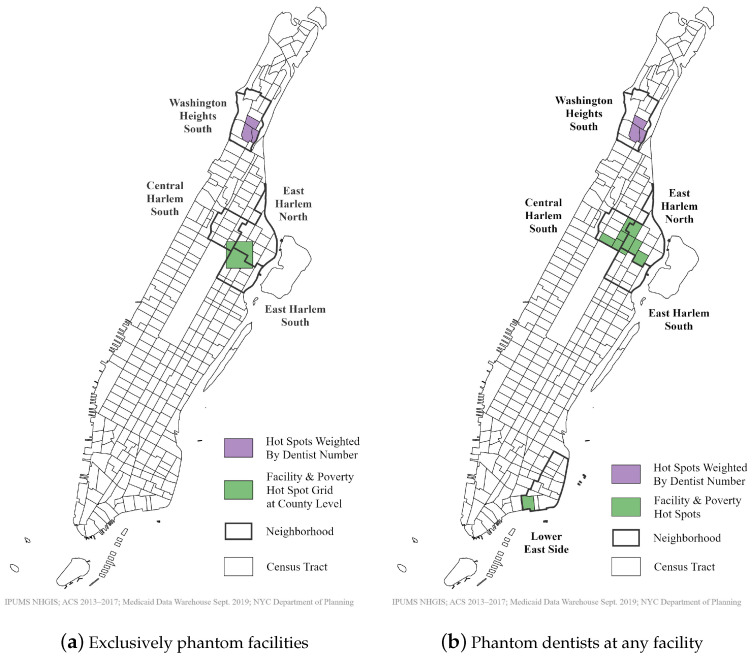
Optimized hot spots of phantom networks and poverty, October 2019.

The spatial association tests found a significant relationship between the presence of poverty and phantom networks. The number of phantom facilities in which all dentists were classified as phantom networks was 54, representing 266 phantom providers. Optimized hot spot analysis found a significant clustering of exclusively phantom facilities and numbers of people in poverty with a 95% confidence level (the confidence levels are not shown in the figures). However, this association was found at the county level rather than at the finer census tract level. This grid clustering fell over the neighborhoods of Central and East Harlem (Figure 3).

When exclusively phantom facilities were weighted by the numbers of phantom dentists present, hot spots occurred in census tracts within the neighborhood of Washington Heights. The confidence level for the clustering of phantom dentists was 95% and the confidence level for poverty ranged from 95% to 99%. The Global Moran’s I Spatial Autocorrelation results indicated a strongly clustered pattern for the numbers of people in poverty per census tract and exclusively phantom networks within 700 feet of each census tract boundary (Table 7). For a Global Moran’s I test, a *p*-value of 0.01 or less and a critical z-score greater than 2.58 indicate a very clustered pattern. Since the z-scores between poverty and the count of exclusively phantom facilities and phantom dentists were 13.64 and 12.27, respectively, there is a less than 1% chance that these patterns are a random occurrence.

Phantom providers were also present at dental facilities where some dentists were verified or eliminated. When adding these numbers to the previous figures, 90 respondent dental facilities had 351 total phantom providers. An interesting pattern occurred when all facilities with phantom providers were analyzed: the spatial association spread to another neighborhood and the previous relationship was preserved (Figure 3). Optimized hot spot analysis found a significant clustering of facilities with phantom dentists and area poverty on the Lower East Side, in addition to Central and East Harlem. The clustering of poverty was strongest in East Harlem and Washington Heights, with some tracts having a confidence interval of 99%.

The clustering of facilities with phantom providers was strongest in one tract within the neighborhood of East Harlem South and one tract on the Lower East Side, having a confidence interval of 95%. However, even though the clustering spread into another neighborhood, the confidence interval was 90% for all other census tracts compared to the grid of 95% for purely phantom facilities at the county level. Nevertheless, when these facilities were weighted by the number of phantom dentists, the confidence interval increased from 95% to 99% in Washington Heights, strengthening the association. The Global Moran’s I Spatial Autocorrelation results indicated a strongly clustered pattern for numbers of people in poverty and all facilities with phantom networks (Table 7). Since the z-score between poverty and the count of facilities was 16.58, and the z-score between poverty and phantom dentists at these offices was 12.34, there is a less than a 1% chance that these patterns are a random occurrence. The addition of any facility with phantom dentists strengthened the z-score of the spatial autocorrelation test for the clustering of facilities and poverty, while the z-score for the numbers of phantom dentists and poverty remained stable. Additionally, evaluating the raw numbers of phantom networks compared to the percentage of people having an income-to-poverty ratio under one [93] by neighborhood hotspot further supported the positive intersection of phantom networks and poverty (Table 8).

There was a weak positive relationship between the numbers of impoverished people and phantom networks according to both the non-spatial Kendall’s tau and Spearman’s rho rank non-parametric correlation tests (Table 9). When analyzing exclusively phantom facilities, the tau score was 0.1141 while the Spearman’s rho was 0.1485. When weighting these offices by the number of phantom dentists, the scores slightly increased to 0.1313 and 0.1768, respectively. When considering any facility with phantom providers, and then weighting these facilities by phantom dentists, the tau and rho scores modestly increased by over 10%, while the *p*-values remained significant. Thus, both spatially and non-spatially, there appears to be a significant correlation between phantom networks and poverty.

## 4. Discussion

### 4.1. Insights and Future Research

This study utilized a novel multidimensional approach to geospatially examine access to general dental care for Medicaid recipients in the borough of Manhattan. We began by analyzing the accuracy of listed dentists in ten managed care organization (MCO) online directories since this is the starting point for the publicly insured to find available dental care. Our hypothesis that MCO directories would present better access to dental care than the New York State (NYS) mandated ratio of one dentist to 2000 Medicaid enrollees within 30 min of public transportation time [66,67] was affirmed. Nevertheless, after applying our exclusion criteria, we were astonished to find the NYS-mandated ratio was still met for verified dentists given that the 2SFCA supply was reduced by nearly 85%, with merely 15–17% of MCO directory providers remaining throughout the census tracts. Since the state’s ratio simply represents the physical count of listed Medicaid dentists who are geographically accessible, it is arguably a limited standard for gauging dental care access and runs the risk of overestimating availability when considering public policy planning.

Current studies have tended to focus on the importance of children’s oral health. However, we chose to tailor our study to examine dental care access for the general population. Thus, our inclusion criteria excluded general dentists who did not treat adults. Our findings were surprising, deviating from the literature that suggests that dental availability is more restrictive for adults compared to children [35,37,74], and that dental providers are unable or unwilling to accept new Medicaid patients [35,37]. In contrast, we found that a low percentage of listed dentists treated children only, and most providers admitted new adult patients with few short-term restraints. These unique findings necessitate future research to inform policy and explore whether any new factors are transforming dental availability for publicly insured adults, particularly where geographic location may play a key role in access to care.

Our novel study fills a gap in the literature concerning dental care phantom networks. The phenomenon of phantom networks, inaccurate provider listings, has been observed in both private and public insurance directories, traversing various health disciplines for over two decades [16,17,18,19,20,21,22,23,24]. We were astonished to find that over two thirds of the dentists surveyed were classified as phantom networks. The leading cause was that dentists were absent from the listed facilities for either months or years prior, and, in some cases, they never worked at the facility at all. This suggests that MCO directories are failing to be updated on a regular basis, as required by law [94]. Further, MCO directories inaccurately list the level of care afforded to the low-income publicly insured, which creates inequities to accessing care from licensed general dentists, and policy efforts may want to provide transparency in online classifications (i.e., student dentists) describing available care. Our research aligns with the available literature scrutinizing inaccurate provider listings that limit accessibility or prevent availability of healthcare [16,17,18,19,20,21,22,23,24].

Researcher recommendations for improving provider directories comprise the following: clearly displaying contact information for the public to report any inaccuracies, regular audits initiated by MCOs, contact with inactive providers by regularly checking claims data, and the creation of a frequently updated global database [19,22,23,24,95]. NYS law requires insurance plans to update online directory provider changes or terminations within 15 days of notification [94]. Therefore, while more research is needed to address the persistence of phantom networks, we believe that the most effective solution would be a real-time database, in which dentists, rather than MCOs, would be required to update accepted insurances and participation status as necessary. Information would then flow firsthand from the dental provider and would provide an audit trail to ensure timely updates.

Nonetheless, finding solutions to phantom networks is complex and entails more than reporting or updating inaccurate MCO directories. NYS contracts with MCOs, who are charged with updating and maintaining the directory of Medicaid dentists [94]. NYS also pays MCOs a monthly capitation rate for each Medicaid recipient irrespective of dental services rendered, thus allowing them to retain any portion of publicly funded monies that are not consumed to meet the insured’s covered services [96]. This may motivate MCOs to undertreat Medicaid recipients [97,98,99,100], as well as endeavor to dissuade enrollees with high maintenance needs from joining [101,102], or offer narrow network plans to limit accessibility. Additionally, MCOs are allowed to set provider payment rates within state contract boundaries, and this may result in low rates and less provider participation [98]. Policymakers may want to institute a refund requirement per insured for unused funds paid to MCOs when no services are rendered during a 12-month period.

Discovering “hidden networks” was an unexpected finding. However, the addition of nine hidden dentists to the verified supply failed to significantly increase access to care. Our research aligns with Haeder, Weimer, and Mukamel [21], who likewise found that hidden physicians failed to significantly improve access to care for those enrolled in marketplace plans, even though their research criteria allowed for plan enrollees to receive care from lower-echelon providers in place of doctors. The topic of hidden networks is under-researched, and future studies are needed to determine both the cause and effect of errors in healthcare data.

We found that requiring patient-centered characteristics significantly decreased dental care accessibility, coinciding with other studies that have postulated that increased standards for network sufficiency reduce provider supply [41,103,104]. To the best of our knowledge, our study is the first to examine whether participating dentists limit the range of clinical services or inquire as to whether dentists place caps on the number of publicly insured patients that they are willing to treat during any timeframe. However, the self-reported low percentages found in this study may have been the result of dental providers’ reluctance to admit to these practices, and the future use of secret shopper inquiries may provide further data for policy planning.

In contrast, we found that extended office hours were limited, and this aligns with scant research that indicates that this time may be insufficient for those working with children, who may lack paid time off for attending dental appointments [47]. Clearly, new studies are essential to determine the temporal range that would be most beneficial for Medicaid patients. Remarkably, new patient acceptance rates among Manhattan practices were high compared to other studies [34,35,36,37]. Few practices served only adult patients, and most respondents identified dentists as full-time providers, which also reflects past studies [37,38,39,41,52]. However, less than half of all verified dental facilities met the entire combination of patient-centered characteristics simultaneously. Future research could explore factors that may motivate dentists to meet patient-centered criteria.

The examination of the relationships between dental access and poverty reveals complex patterns that are tied to the urban structure and market choice. The spatial Multivariate Local Geary test revealed a positive relationship between dental care access and poverty numbers for most Manhattan census tracts. The test is based on the relationship between a census tract and its neighboring tracts. For a tract with a high poverty rate, if Medicaid dental access is also high in the surrounding neighborhood encompassing the six closest tracts, relative to randomly chosen tracts from the borough, the test will be positive. In contrast, the non-spatial Kendall’s and Spearman’s rank correlation tests found a weak negative relationship, without referring to any neighboring tracts. While these results may seem contradictory, they exactly reflect the notion that dental facilities are less likely to be located within high-poverty tracts, and instead are spatially scattered within their nearest neighbors. Dental providers, therefore, can offer services to Medicaid recipients in nearby impoverished census tracts, while being located in the more affluent tracts to maximize profits.

The literature on location decisions in the medical marketplace supports the notion that physicians and dentists generally choose to establish their practices in more affluent areas [105,106,107,108,109,110]. This location behavior may serve to attract private patients to offset the low reimbursement rates of Medicaid [28], hence counterbalancing costly student loan debt [111,112]. Location decisions may be financially motivated rather than needs-based, thus leading to an inequitable distribution of health providers. In fact, economic segregation between people of low and high incomes has been increasing in American urban areas for over 30 years, and the New York metropolitan area is among the most economically segregated in the nation [113,114]. To our knowledge, this is the first study analyzing the relationship between poverty and dental care access utilizing spatial statistics, and more research is needed to examine the effects of such spatial contexts in other intraurban areas.

The endeavor to use geographic methods to determine whether the phenomenon of phantom networks occurs in urban neighborhoods with the greatest poverty is novel. Both spatial and non-spatial tests revealed a significant correlation between people in poverty and the presence of phantom networks. The fact that both tests became more significant with a greater sample size between exclusively phantom facilities and facilities with any number of phantom providers suggests that the correlation is robust [115]. The presence of phantom networks in impoverished urban areas warrants further research to uncover the root causes of these phenomena and discover if they occur in other disadvantaged intraurban areas. Additional demographic variables, such as race or immigration status, may also be associated with phantom networks, and future research is needed to expand the knowledge about these topics.

### 4.2. Limitations

This study should be interpreted in consideration of several important limitations. Data collected from dental facilities were subject to self-reporting bias. Medicaid providers were estimated for eight of the 118 respondent facilities where dentists’ names had been omitted from MCO directories, and this may have over- or under-estimated the number of Medicaid dentists. The 2SFCA method measured spatial accessibility from the perspective of general Medicaid acceptance as opposed to each individual MCO plan, which likely overestimated accessibility when narrow networks were present. When estimating the percent increase in the Medicaid population between 2017 and 2019, the increase was quantified as uniform instead of tangible across the census tracts.

The border-crossing problem may have over- or under-estimated dental care access because nearby boroughs were eliminated; consequently, neighboring dental facilities and competing populations were omitted from the 2SFCA. The surveying of dental facilities was conducted from November to December 2019 to test MCO directories accessed in October of the same year, and the MCOs may have subsequently updated their databases. It should also be noted that this research took place shortly before the COVID-19 pandemic, and this event may have subsequently affected dental care access for Medicaid recipients. Future research should address how social distancing, healthcare shortages, and other healthcare disruptions may have impacted the utilization of dental care by Medicaid recipients and the availability of dentists.

## 5. Conclusions

The most salient result of our novel study is that phantom networks mask disparities to dental care access and create inequities for a low-income and underserved population. Inaccurate MCO listings vastly reduce the Manhattan Medicaid dental supply, and this phenomenon hinders the vulnerable from finding essential dental care access, which is a concern in policy decisions. Measuring the availability of multiple patient-centered characteristics diminished the dental supply further due to dental provider restrictions, and this topic is relevant to understanding the service utilization of the publicly insured. Our analysis to measure the spatial association between poverty and dental care access is unique and revealed a complex relationship affecting the location decisions of dentists. Dental providers tend to be located in affluent communities near to impoverished neighborhoods to allow for a mixture of both private and publicly funded patients, serving both communities, thus ensuring profitability. This is further supported by the original finding of a positive correlation between phantom networks and areas of greater poverty. The combination of measuring dental availability and accessibility utilizing geographical methods to assess dental care access for the underserved offers new insights and data regarding an under-researched topic.

## Figures and Tables

**Table 1:** Ten most common Medicaid MCO plans in NYC.

1	Affinity Health Plan
2	AmidaCare
3	Empire Blue Cross Blue Shield/Amerigroup HealthPlus
4	Emblem Health Insurance Plan of Greater New York
5	Fidelis Care
6	HealthFirst Prepaid Health Services Plan
7	MetroPlus Health Plan/MetroPlus Health Plan Special Needs
8	United Healthcare Plan of New York
9	Visiting Nurse Services Choice Plan
10	Wellcare of New York

**Table 2:** Survey responses of facilities and dentists in MCO directories.

MCO-Listed Facilities: 259			
Respondents	118	Response Rate	45.6%
MCO-Listed Dentists: 868			
Listed Facility Dentists	509	Percent of MCO Dentists	58.6%
Verified Dentists	70	Verified Percent	13.8%
Phantom Dentists	351	Phantom Percent	69.0%
Eliminated Dentists	88	Eliminated Percent	17.3%
Hidden Dentists: 9			
New Verified Total	79	Verified Percent Increase	12.9%

**Table 3:** Reasons for eliminated status.

Rank	Reason for Elimination	Count	Percent
1	Dentists serve children only	49	9.6%
2	Dentists work less than 20 h per week	39	7.7%
88 Eliminated Dentists out of 509 Listed Dentists			

**Table 4:** Reasons for phantom networks.

Rank	Reason for Phantom Classification	Count	Percent
1	Dentists do not work at listed facilities	113	22.2%
2	Dentists serve a limited population	92	18.1%
3	Licensed dentists do not provide services	82	16.1%
4	Dentists are not general providers	47	9.2%
5	Dentists refuse the listed insurance	17	3.3%
351 Phantom Dentists out of 509 Listed Dentists			

**Table 5:** One-sample *t*-test results.

MCO Directory Providers (Figure 1a)	
t = 46.0	Data frame = 270
*p*-value < 2.2 × 10^−16^	Mean = 0.0032 (1:315)
Alternative hypothesis H_0_:	True mean > 5 × 10^−4^ (1:2000)
95% confidence interval:	0.0031, *∞*
The test rejects the hypothesis that 2000 Medicaid patients have access to less than one	
general dentist.	
Verified Providers (Figure 1b)	
t = 2.1	Data frame = 270
*p*-value = 0.01646	Mean = 0.00052 (1:1927)
Alternative hypothesis H_0_:	True mean > 5 × 10^−4^ (1:2000)
95% confidence interval:	0.0005, *∞*
The test rejects the hypothesis that 2000 Medicaid patients have access to less than one	
general dentist.	
Patient-Centered Dentists (Figure 1c)	
t = −73.6	Data frame = 270
*p*-value = 1	Mean = 0.00022 (1:4587)
Alternative hypothesis H_0_:	True mean > 5 × 10^−4^ (1:2000)
95% confidence interval:	−∞, 0.00021
The test cannot reject the hypothesis that 2000 Medicaid patients have access to less than one	
general dentist.	

**Table 6:** Dental facilities fulfilling patient-centered criteria.

Patient-Centered Facility Criteria	Yes	No	Percent
Accept New Patients	46	4	92%
Serve All Ages	47	3	94%
Provide Full Services	46	4	92%
Offer Extended Hours	35	15	70%
Work Full-Time	35	15	70%
Place No Caps on Patients	49	1	98%
All Criteria	20	30	40%
50 verified facilities representing 79 verified dentists			

**Table 7:** Global Moran’s I Spatial Autocorrelation of poverty numbers and phantom networks within 700 feet of Manhattan census tract boundaries.

Poverty Numbers and Count of Exclusively Phantom Facilities	
Moran’s Index: 0.44	
z-score: 13.64	Clustered Pattern
*p*-value: 0.00	
Poverty Numbers and Count of Phantom Dentists at Exclusively Phantom Facilities	
Moran’s Index: 0.37	
z-score: 12.27	Clustered Pattern
*p*-value: 0.00	
Poverty Numbers and Count of Any Facility with Phantom Dentists	
Moran’s Index: 0.53	
z-score: 16.58	Clustered Pattern
*p*-value: 0.00	
Poverty Numbers and Count of Phantom Dentists at Any Facility	
Moran’s Index: 0.38	
z-score: 12.34	Clustered Pattern
*p*-value: 0.00	

**Table 8:** A comparison of poverty rates and number of phantom networks by neighborhood where optimized hot spots occurred.

Neighborhood Hot Spot	Poverty Percent	Purely Phantom Facilities	All Facilities with Phantom Dentists	Dentists at Purely Phantom Facilities	Phantom Dentists at All Facilities
East Harlem North	38.2%	6	9	-	-
Lower East Side	30.1%	-	5	-	-
East Harlem South	28.7%	6	7	-	-
Central Harlem South	24.6%	3	10	-	-
Washington Heights South	24.6%	-	-	100	100
Neighborhood Average	19.5%	3	5	15	19

Source: Poverty data were taken from the American Community Survey (ACS) 5-year estimates from 2013–2017.

**Table 9:** Correlation between phantom networks and poverty.

Phantom Network	Kendall’s Tau	*p*-Value	Spearman’s Rho	*p*-Value
Exclusively Phantom Facilities *	0.1141	0.0161	0.1485	0.0144
Number of Phantom Dentists	0.1313	0.0037	0.1768	0.0035
Any Facility with Phantom Dentists **	0.2329	3.913 × 10^−7^	0.3072	2.472 × 10^−7^
Number of Phantom Dentists	0.2300	1.441 × 10^−7^	0.3173	9.395 × 10^−8^

* There were 54 exclusively phantom facilities with 266 phantom providers. ** There were 90 dental facilities with 351 phantom providers.

## Data Availability

The data presented in this study are available on request from the first author.

## Supplementary Materials

The following are available online at https://www.mdpi.com/article/10.3390/ijerph182312383/s1, Figure S1: Medicaid Dental Supply in Manhattan Census Tracts, Table S1: Respondent Dentists by Insurance Plan, Table S2: Classification of Dentists by Insurance Plan, Table S3: Effect of Hidden Providers on Verified Supply by Insurance Plan and the Survey Questions.
