# Supplementary material for: How Phantom Networks, Provider Qualities, and Poverty Sway Medicaid Dental Care Access: A Geospatial Analysis of Manhattan
Source: Int J Environ Res Public Health. 2021 Nov 25;18(23):12383. doi: 10.3390/ijerph182312383 (PMC8656799; doi:10.3390/ijerph182312383)
Supplement: Supplementary file 1 [file ijerph-18-12383-s001.zip › supplementary_material/supplementary_material.pdf]

SUPPLEMENTARY MATERIAL

EXTRA MEDICAID DENTAL SUPPLY MAPS

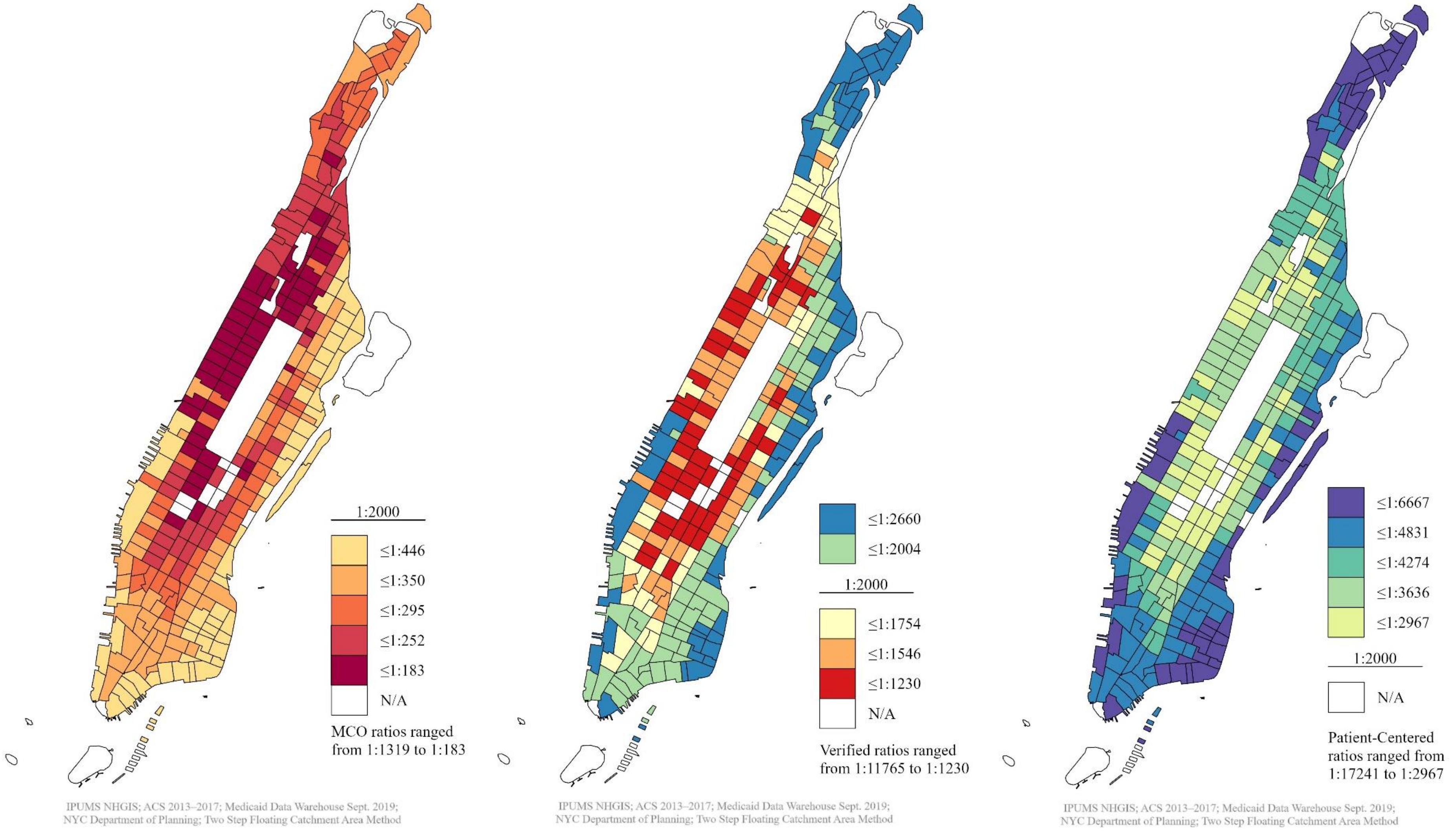

Figure S1: Medicaid Dental Supply in Manhattan Census Tracts, October 2019 **(A)** MCO Directory-Derived, **(B)** Verified, and **(C)** Patient-Centered.

BREAKDOWNS BY INSURANCE PLAN

Table S1: Respondent Dentists by Insurance Plan

MCO Insurance	Facilities	Respondent Facilities	Response Rate	Listed Dentists	Respondent Dentists	Dentist Response Rate
Affinity	191	83	43%	561	349	62%
AmidaCare*	47	25	53%	142	89	63%
Emblem Health	3	0	0%	3	0	0%
EmpireBCBS	97	42	43%	239	109	46%
Fidelis*	16	8	50%	57	37	65%
Healthfirst	203	90	44%	641	391	61%
MetroPlus	85	44	52%	232	120	52%
UnitedHealth	121	57	47%	255	129	51%
VNSC*	5	2	40%	17	12	71%
WellCare	9	6	67%	42	29	69%

Note: The number of dentists includes the same dentist working at different facilities.
*These insurance lists had facilities without any providers listed. Therefore, the number of dentists was estimated from information provided by other MCO lists.

Table S2: Classification of Dentists by Insurance Plan

MCO Insurance	Verified Dentists	Percent	Phantom Dentists	Percent	Eliminated Dentists	Percent
Affinity	40	11%	243	70%	66	19%
AmidaCare*	11	12%	55	62%	23	26%
Emblem Health	0	0%	0	0%	0	0%
EmpireBCBS	30	28%	56	51%	23	21%
Fidelis*	9	24%	27	73%	1	3%
Healthfirst	59	15%	270	69%	62	16%
MetroPlus	39	33%	58	48%	23	19%
UnitedHealth	23	18%	72	56%	34	26%
VNSC*	0	0%	11	92%	1	8%
WellCare	2	7%	20	69%	7	24%

Note: The number of dentists includes the same dentist working at different facilities.
*These insurance lists had facilities without any providers listed. Therefore, the number of dentists was estimated from information provided by other MCO lists.

Table S3: Effect of Hidden Providers on Verified Supply by Insurance Plan

MCO Insurance	Facilities	Hidden Facilities	Verified Dentists	Hidden Dentists	New Verified Dentists	Percent Increase
Affinity	5	0	54	6	60	11%
AmidaCare*	7	5	89	14	103	16%
Emblem Health	30	30	0	44	44	N/A
EmpireBCBS	9	6	23	11	34	48%
Fidelis*	43	42	1	63	64	6300%
Healthfirst	7	5	62	8	70	13%
MetroPlus	6	1	23	6	29	26%
UnitedHealth	10	3	34	11	45	32%
VNSC*	10	10	0	17	17	N/A
WellCare	13	13	2	23	25	1150%

Note: The number of dentists includes the same dentist working at different facilities.
*These insurance lists had facilities without any providers listed. Therefore, the number of dentists was estimated from information provided by other MCO lists.

Forty facilities from the combined insurance lists of Fidelis, AmidaCare and Visiting Nurse Services were missing dentist names. Thirty-two of these facilities also occurred in other MCO directories where dental provider names were included. Thus, the dentists listed as accepting Medicaid in these other directories were assumed to also accept the insurances in which the dentists were omitted. However, eight of the facilities without provider names appeared only in the Fidelis directory. Therefore, the number of dentists was estimated using the average number of dentists working at surrounding facilities located within a one-mile radius of the eight individual offices with the Find by Location tool in ArcGIS Pro.

Results

Breakdown of dentists by individual Medicaid Managed Care Organization (MCO) insurances revealed some interesting facts. The broadest provider networks based on MCO directories were Healthfirst and Affinity, whereas Emblem Health, Fidelis, Visiting Nurse Services and WellCare offered a narrow network of dentists. Dental provider response rates ranged from 71% for Visiting Nurse Services to 0% for Emblem Health (Table S1). The MCO with the most verified dentists was MetroPlus at 33% (Table S2). The MCO with the least verified providers was Visiting Nurse Services, in which most of the listed dentists that responded were phantom networks (92%). MetroPlus had the least phantom dentists at 48%. AmidaCare and UnitedHealth had the most eliminated dentists at 26%, while Fidelis had only 3% of their dentists classified as eliminated providers.

It was found that some dentists listed in a limited number of MCO directories did in fact accept additional MCO insurance plans, even though this data was unlisted in the plan directories (Table S3). These providers are designated as hidden networks. The MCOs that provided narrow

networks were most affected by the addition of hidden providers. The percent increase of verified dentists significantly exceeded 100% for Fidelis and WellCare, ranging from 6,300% to 1,150%. The percent increase for Emblem Health and Visiting Nurse Services was incalculable since neither plan had any verified providers before the addition of hidden networks. Fidelis had the most hidden dentists with 63 new providers, followed by Emblem Health with 44 dentists, WellCare with 23 dentists, and Visiting Nurse Services with 17 new dentists. Insurances providing broader networks had a lower number of hidden dentists and a lower percent increase of verified providers.

Discussion

All MCOs examined in this study contained phantom networks. The MCOs with the greatest number of phantom dentists were Healthfirst, Affinity, and UnitedHealth. These MCOs provided broader networks, and they may have been motivated to list numerous dental providers to encourage potential insureds to enroll in their plan, even though there are fewer providers participating in the plan than advertised, and this aligns with Daw (2002). In addition, the state pays MCOs a monthly capitation rate or fixed fee for each Medicaid enrollee regardless if any dental services are rendered, even if a Medicaid recipient is unable to find a participating dentist after several attempts at using an MCO directory with inaccurate listings (Medicaid and CHIP Payment and Access Commission (MACPAC) 2019). MCOs may be motivated to use narrow networks to impede Medicaid recipients from finding an available dentist to avoid paying for covered services. Hidden networks may well correspond to narrow networks, such as those displayed in the directories for EmblemHealth, Fidelis, Visiting Nurse Services and WellCare, since narrow networks contained the most hidden networks. However, this innovative topic requires further research to determine the links between the two networks.

References

Daw, Jennifer. 2002. “Fighting the Phantoms of Managed Care: Legislation in California and a Legal Case in Virginia Address Managed Care’s Practice of Maintaining Phantom Provider Panels.” *Journal of Monitor on Psychology* 33 (2): 21. https://www.apa.org/monitor/feb02/phantoms.html.

Medicaid and CHIP Payment and Access Commission (MACPAC). 2019. “Managed Care’s Effect on Outcomes.” Washington, DC. https://www.macpac.gov/subtopic/managed-cares-effect-on-outcomes/.

SURVEY QUESTIONS

Questions for Dental Offices and Dentists

General Interview Questions:

Consent: Before participating in this survey, I have understood and agree to the terms of the Oral and Internet Consent Form provided by the principal investigator of this study.

Note: For every question, please provide only information about licensed dentists at your facility. Please do not include information about dental assistants, dental hygienists or student dentists who have not yet obtained a license who may be fulfilling residency requirements, etc. This study only concerns licensed dentists.

(A link to the Qualtrics software version of the survey was provided here with the password.)

Note: The format of the interview questions over the phone will be different from the format of the questions on Qualtrics; however, the content of the questions is the same.

1. What is the address of this dental office?

2. What is the actual business name of this dental office?

3.1 Is this address located within the borough of Manhattan?

3.2 Is this the only address upon which this practice is based?

If the respondent answers *no* to Question 3.1, and *yes* to Question 3.2, the interview will be concluded.

3.3 Are any of these other facilities located in the borough of Manhattan?

If the respondent answers *no* to Question 3.1, Question 3.2 and Question 3.3, the interview will be concluded.

3.4 Please provide any addresses of your other facilities in the borough of Manhattan, including their business names. Any facilities outside the borough of Manhattan are beyond the scope of this study, so please do not list them.

Once the interview is completed, please answer these questions for each alternative facility, or please provide information for contacting the other offices.

4. Do any licensed dentists in your office provide general dentistry services?

If the respondent answers *no* to Question 4, the interview will be concluded.

5.1 Which of the following Medicaid Managed Care Organization (MCO) Plans listed below are accepted by your office? If none of these plans are accepted by any licensed dentist in your office, please indicate “No licensed dentist in our office accepts any of these plans."

- Affinity Health Plan
- Amida Care
- Fidelis Care
- HealthFirst Prepaid Health Services Plan
- Empire Blue Cross Blue Shield Amerigroup HealthPlus
- EmblemHealth Insurance Plan of Greater New York
- MetroPlus Health Plan Special Needs
- MetroPlus Health Plan
- United Healthcare Plan of NY
- Visiting Nurse Services Choice Plan
- Wellcare of New York
- No licensed dentist in our office accepts any of these plans

If the respondent answers *No licensed dentist in our office accepts any of these plans* to Question 5.1, the interview will be concluded.

If the respondent answers *yes* to Question 5.1, then:

5.2 Please provide the names of your licensed dentists who accept each plan and whether each licensed dentist is accepting any new patients under each plan.

5.3 Do the majority of your licensed dentists (i.e. more than half) serve all Medicaid populations, or do the majority serve only certain groups such as: children or elderly only?

If there is an equal number of dentists for both criteria, please provide the number of licensed dentists who serve all Medicaid patients and how many serve only certain groups.

5.4 Do the majority of your licensed dentists (i.e. more than half) offer all possible dental services to Medicaid patients, or are patient services limited to treatments such as: dental cleanings and x-rays only?

If there is an equal number of dentists for both criteria, please provide the number of dentists who offer a full range of services and how many dentists offer only limited services.

5.5 What are the days and hours of operation for this facility?

5.6 Do the majority of your licensed dentists (i.e. more than half) work 40 hours or more per week (i.e., full-time) treating patients, or do the majority work less than 40 hours per week (i.e., part time) treating patients?
If there is an equal number of dentists for both criteria, please provide the number of licensed dentists who work full-time treating patients and how many licensed dentists work part time treating patients.

5.7 Do the majority of your licensed dentists (i.e. more than half) limit the number of Medicaid patients seen per day or within any other time frame, or do the majority place no limits on the number of Medicaid patients seen within any time frame?

If there is an equal number of dentists for both criteria, please provide the number of licensed dentists who limit the number of Medicaid patients they are willing to see under any time frame, and how many licensed dentists are willing to see any number of Medicaid patients under any time frame.

For example, “Dr. X only accepts one Medicaid patient per day. Dr. Y has no limits on the number of Medicaid patients seen per day. Dr. Z accepts three Medicaid patients per week.”

Once Question 5.7 is answered, the interview is concluded.

Follow-up: If there is any missing information from this interview, may I follow up with you later in order to receive any necessary data? If yes, please provide an email or telephone number.
