# Supplementary figures and images for: How Phantom Networks, Provider Qualities, and Poverty Sway Medicaid Dental Care Access: A Geospatial Analysis of Manhattan
Source: Int J Environ Res Public Health. 2021 Nov 25;18(23):12383. doi: 10.3390/ijerph182312383 (PMC8656799; doi:10.3390/ijerph182312383)

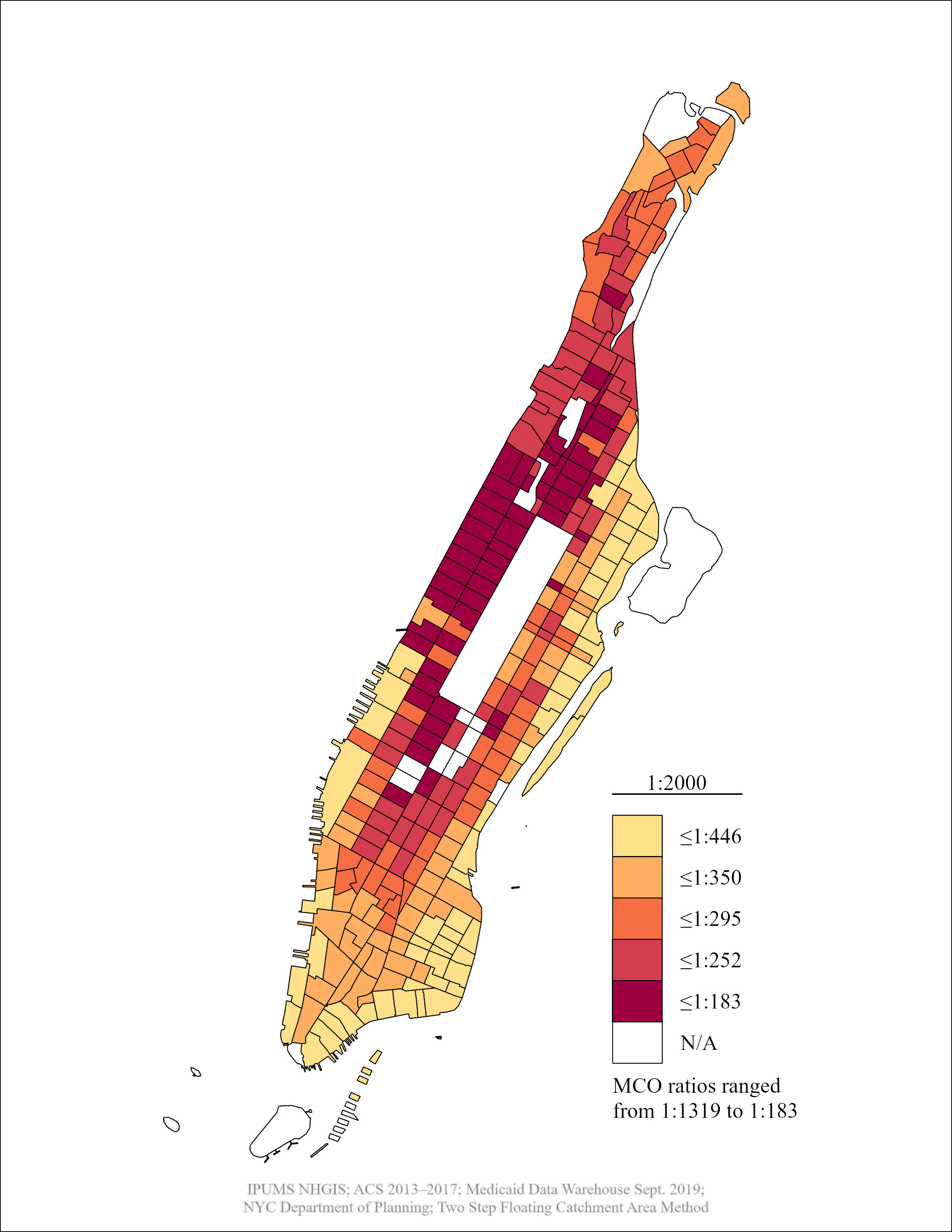

Supplement: Supplementary file 1 [file ijerph-18-12383-s001.zip › supplementary_material/Q1_2SFCA_Separate.jpg]

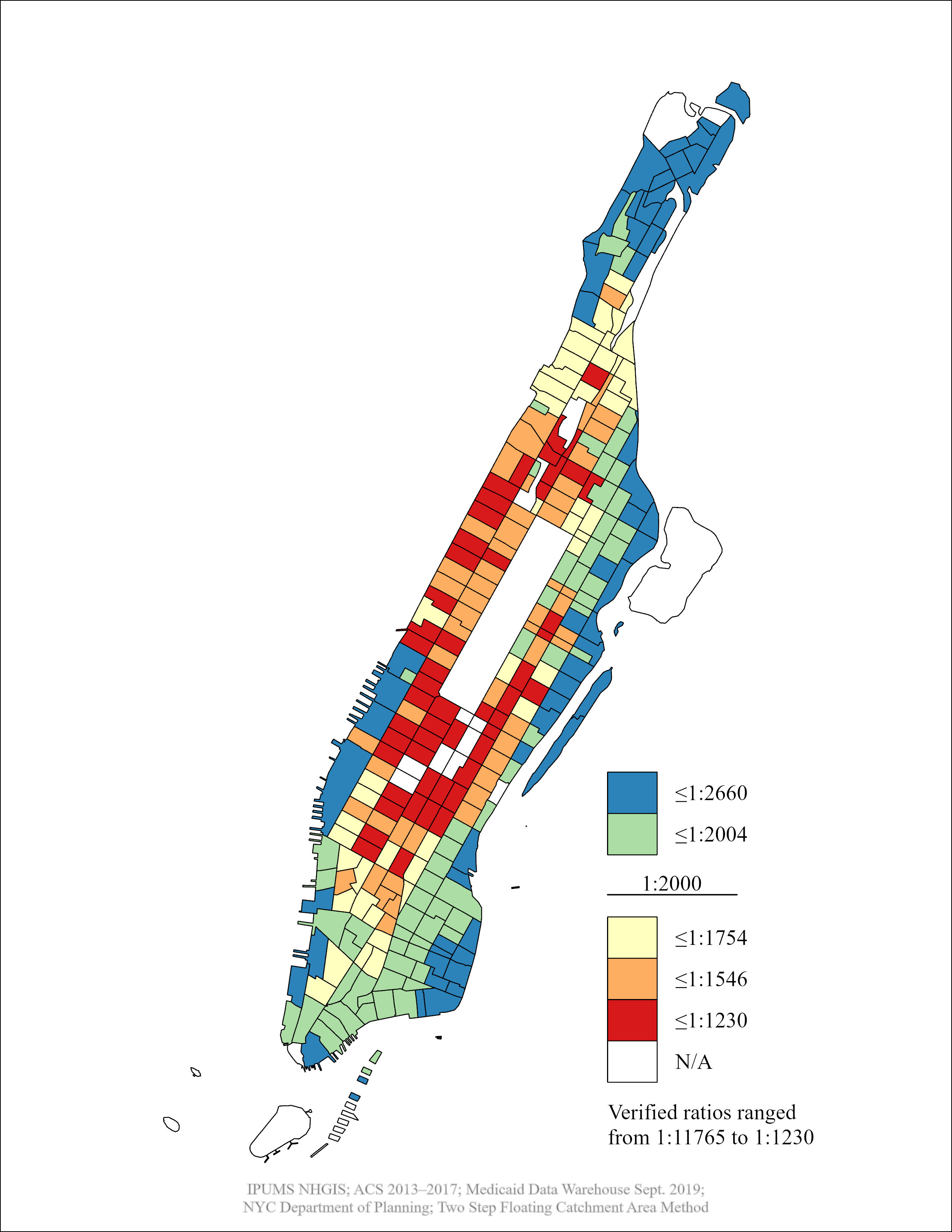

Supplement: Supplementary file 1 [file ijerph-18-12383-s001.zip › supplementary_material/Q2_2SFCA_Separate.jpg]

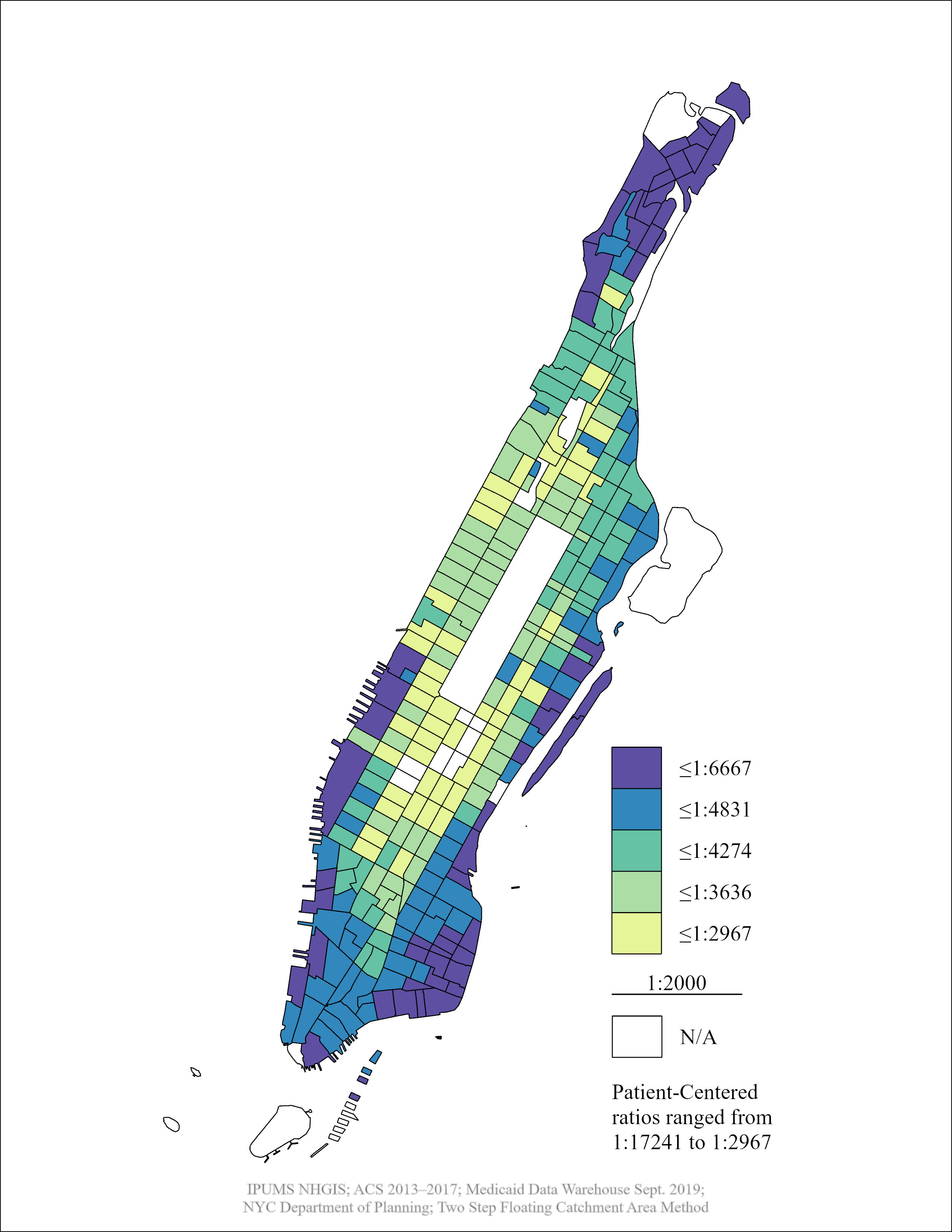

Supplement: Supplementary file 1 [file ijerph-18-12383-s001.zip › supplementary_material/Q3_2SFCA_Separate.jpg]
